# Promoting Effect of L-tyrosine Supplement on New Zealand Rabbit Bucks' Performance and Reproduction Through Upregulation of Steroidogenic Markers

**DOI:** 10.3389/fvets.2020.00605

**Published:** 2020-10-14

**Authors:** Shawky Mahmoud, Michel Saad, Ahmed M. El-Shehawi, Foad Farrag, Mahmoud M. Abo Ghanima, Mahmoud A. O. Dawood, Mustafa Shukry

**Affiliations:** ^1^Department of Physiology, Faculty of Veterinary Medicine, Kafrelsheikh University, Kafrelsheikh, Egypt; ^2^Department of Biotechnology, College of Science, Taif University, Taif, Saudi Arabia; ^3^Department of Genetics, Faculty of Agriculture, Alexandria University, Alexandria, Egypt; ^4^Department of Anatomy, Faculty of Veterinary Medicine, Kafrelsheikh University, Kafrelsheikh, Egypt; ^5^Animal Husbandry and Animal Wealth Development Department, Faculty of Veterinary Medicine, Damanhour University, Damanhour, Egypt; ^6^Department of Animal Production, Faculty of Agriculture, Kafrelsheikh University, Kafrelsheikh, Egypt

**Keywords:** L. tyrosine, New Zealand rabbits, testosterone, steroidogenesis markers, summer, fertility

## Abstract

Delayed puberty and lower fertility are among the most challenging concerns in rabbit development during the summer season. This study was, therefore, aimed at enhancing male NZ rabbits' performance by using L-tyrosine. Thirty male, New Zealand rabbits, were employed for this purpose at the age of 60 days. Rabbits were divided accidentally into two groups: a control group and another treated with L-tyrosine (100 mg/kg body weight). After 4 weeks, three bucks of each group were assassinated. A comparable oral dose of L-tyrosine was administered to half of the treated group left untreated during the second half. Weekly blood samples were assembled from each group for testosterone, T3, and T4 hormone testing. The results showed that body weight and serum testosterone, T3, and T4 increased exponentially with increasing age in both groups. L-tyrosine contributed to another vital rise in dose-dependence than control, in bodyweight, GSI, and testosterone, T3, and T4. At the end of the third month, tests fell in the scrotum, compared to 2 weeks before in the L-tyrosine group. In the middle of the fourth month, the semen evaluations were first carried out for the L-tyrosine group and 1 month after for the control group. L-tyrosine has contributed to a substantial upsurge in semen quality and motility, and abnormalities have reduced dramatically (*P* < 0.01). The L-tyrosine-treated group showed significantly increased mRNA expression of steroidogenesis markers STAR, CYP11A1, and 3B-HSD. Besides, free sperm in the seminiferous tubular lumen was discovered at the end of the third month. Nevertheless, it achieves only in control of the spermatocyte stage. The research suggests that L-tyrosine supplements promote puberty and improve male New Zealand rabbit fertility during high-temperature periods in the year.

## Introduction

The testes are hidden inside the abdominal cavity when a young rabbit is born. They fall in the scrotum at around 3–4 months of age, abruptly before puberty (1). The male rabbit's puberty is described as when it first shows the endocrine testicular function or the first time that a male can deliver sperm (2). Puberty was demonstrated a month before most rabbits reached puberty, and an age difference existed between rabbits of different races. White bucks in New Zealand reached puberty at the age of 5 months (3). Rabbit testicular growth follows a sigmoid curve that grows exponentially during puberty (4). It was reported that the weight of each testis in New Zealand white rabbits was 3.08 ± 0.1 g at ~ 5 months of age (5).

In New Zealand, spermatogonia mitosis first happened at 8 weeks of life, and the Leydig cell growth predated the initiation of spermatogenesis (6, 7). Sperm cells were between 9 and 12 weeks of age; sperm maturation was observed between 13 and 14 weeks, and at the end of the fourteenth week, sperm was found in the tubular lumen (8).

Regular rhythms in amino acid metabolism have discovered a correlation between food intake and neurotransmission (4). L-tyrosine, an amino acid of catecholamine, affects thyroid hormones and protein (9, 10). Dopamine is one of the hypothalamic catecholamines regulating prolactin secretion and helps in growth hormone activation (11, 12). L-tyrosine is considered acceptable for all animal species when supplemented for non-food animals (13). The puberty age in rats was decreased by external L-tyrosine (10) and increased pulse frequency in growing lambs (11). It also accelerated maturity and improved the body weight gain of female New Zealand rabbits (14, 15). L-tyrosine also enhances testosterone levels and semen quality of bulls (16). Also, cows treated with one or two doses of L-tyrosine (50 gm/bull calves) induced an improvement in growth and yield (17).

One of the issues faced in the rearing of rabbits is the reduced breeding success in the high-temperature season. The purpose of this report, consequently, was to analyze the influence of oral given L-tyrosine on the creation of thyroid and testosterone in puberty and hormonal levels, along with semen quality and steroidogenesis markers in New Zealand bucks during the warm season of the year.

## Materials and Methods

### Ethical Statement

The committee approved animal maintenance and used the Faculty of Veterinary Medicine's experimental procedure, Kafrelsheik University, Egypt, which authorized the present study (KVM018/2017; May 2017).

### Experimental Animals

This experiment took place from March to July. Thirty male NZ rabbits weighing 950–1,050 g at 45–55 days were used in the current study. After a week, they acclimatized. Rabbits were allotted to two groups, control (11 bucks) and treated groups (19 bucks). Orally, L-tyrosine was given at 100 mg/kg b.wt (Serva, Heidelberg, Germany) (18) at about 8 weeks of age. The following 4 weeks after L-tyrosine was orally administered, three male rabbit bucks from the standard control and treated group were killed. The testes were weighed and prepared for histopathological technique and transcriptomic analysis. L-tyrosine's common oral dosage was given to half of the treated group (*N* = 8), while the other half remained untreated. After another 4 weeks, three bucks from each group were killed. Semen selection and assessment were applied to the remaining bucks (five in each group). The rabbits were fed a diet of 16.76% protein, 2.36% ether extract, 12.2% fiber, and 2,600 k digestible energy over the experimental period. Durable energy consumption calories/kg and vitamin and mineral nutritional needs were met (19). Sigma-Aldrich (St Louis, MO, USA) provided all the reagents.

### Sampling

Blood samples were obtained each week from the ear vein of the rabbits for two and half months from the first day of orally given L-tyrosine. Serum blood samples were utilized for evaluation of the thyroid and testosterone. Three male rabbit bucks from the control and treated groups were executed following four and 8 weeks since the beginning of the experiment. A portion of each buck's testes were preserved for histological studies in 10 % neutral buffered formalin. Simultaneously, another portion of the RT-PCR analysis was dissected and frozen at −80°C.

### Gonadosomatic Index

Tests were externalized and weighed to calculate the gonad somatic index.

GSI = Testes weight (g) × 100/Total body weight (g).

### Histological Study

Testicular tissue samples taken four and 8 weeks from the start of the trial for histological parts were fixed in 10 percent neutral formalin. Sections of 5–7 microns in thickness were prepared with hematoxylin and eosin-stained (20) for spermatogenesis alterations.

### Hormonal Assays

Serum testosterone measurements were assayed using (Active® testosterone RIA DSL-4000) after 60 min of incubation at 37°C, then 50 μL was transferred to test tubes of polypropylene, and 500 μL T [I125] was added. The tubes were rinsed, inverted for 5 min on paper towels, and measured on the gamma counter. Guidelines for standard and control followed the same protocol. The coefficient of intra-assay variation was 7.9–9.6 % and the coefficient of inter-assay variation was 8.4–9.1 %, according to Reiter and Grumbach (21).

Serum thyroxine (T4) and triiodothyronine (T3) were measured using (Active® thyroxine DSL 3200) and (Active® T3 RIA DSL 3100), respectively, and assayed by RIA kits bought from the Diagnostic System Laboratories Inc. (DSL). We focused on the existence of unique antibodies adhering to the internal surface of the propylene tube. Rabbit hormone-stripped serum was used for the standard total T4, T3 curves, inter-assay, and intra-assay variance coefficients ranging from 7.1 to 7.4 and from 2.9 to 5.1 %, respectively, following Engler and Burger (22) and Yalow and Berson (23).

### Semen Assessment

Beginning with the initial L-tyrosine dose, rabbit bucks (digital palpation) were noticed and examined every day for the fall of the testes into the scrotum. Semen ejaculate assemblage tests were done for all the bucks with the use of an artificial vagina and/or live doe (determine reaction point in time). Semen ejaculate was gathered in the mornings twice a week. Following regular semen collection, each semen ejaculate was transferred immediately to a 35°C water bath, and different semen analysis was carried out after the gel was removed. The quantity of the ejaculate was measured by the graduated tube array. Mass sperm motility (0–5), individual sperm motility %, and sperm cell anomalies were evaluated by following Salisbury et al. (24). The sperm concentration was measured using the Neubauer hemocytometer. The live sperm was estimated by eosin-nigrosine stained films (25). Semenal ejaculate assessment was performed for 4 months following the initial dose of L-tyrosine.

### Gene Expression

Using RT-PCR, a separate expression of the testicular genes was determined. In brief, the TRIzol reagent (Invitrogen, Life Technologies, Carlsbad, CA, USA) was used to extract total RNA from around 100 mg of testicular tissue. Quantitative Nanodrop RNA samples of 1.8 or more A260/A280 were used to synthesize DNA using a cDNA synthesis package (Fermentas, Waltham, MA, United States). To amplify cDNA, the SYBR Green master mix and the primers listed in Table 1 with glyceraldehyde-3-phosphate dehydrogenase (GAPDH) as a household gene were added. Data on amplification were analyzed using 2^−ΔΔ^ methods (26).

**Table 1:** Primer sequences.

**Gene**	**Forward primer (5^**′**^-3^**′**^)**	**Reverse primer (5^**′**^-3^**′**^)**	**Product size**	**Accession number**
STAR	F	ACCAAACTCACTTGGCTGCT	270 bP	XM_017350352
	R	ACACTATTGTCCCACTGCCG		
CYP11A1	F	TACCATTTGGCTGAGGAGAAGG	210 bP	S59219
	R	GTACTCGTGATAGGCGACCC		
3B-HSD	F	TGGGACACAGTTCCTGGTCT	102 bP	XM_008248712
	R	GCCTCATATGGGGTGTCCTC		
GAPDH	F	AGACACGATGGTGAAGGTCG	164 bP	L23961
	R	TGCCGTGGGTGGAATCATAC		

*STAR, steroidogenic acute regulatory protein; CYP11A1, cytochrome P450 cholesterol side-chain cleavage enzyme; 3B-HSD, hydroxy-delta-5-steroid dehydrogenase, 3 beta-and steroid delta-isomerase 7; GAPDH, glyceraldehyde-3-phosphate dehydrogenase*.

### Statistical Analysis

Using version 23.0 of the SPSS program (SPSS Inc., Chicago, IL, USA), statistical analysis of the collected data was carried out using one-way ANOVA, the multiple comparison tool used by the Duncan multi-range Tukey posthoc trials. Described statistical significance was *P* < 0.05.

## Results

One L-tyrosine dose, as displayed in Table 2, caused a substantial change in overall body weight in contrast with control. By using a second dose of L-tyrosine, those parameters and GSI were further increased. With advancing age in the control rabbit group, serum testosterone (ng/ml) was steadily raised. As against the control group, L-tyrosine caused a substantial dose-dependent rise in serum testosterone levels over the study duration (Table 3). T3 and T4 serum levels in all progressive age groups were markedly increased after 4 months and decreased afterward. L-tyrosine, orally administered, resulted in a marked surge in T3 and T4 quantities compared with to control group (Tables 4, 5).

**Table 2:** Total body weight, testes weight, and GSI of New Zealand bucks treated with L. tyrosine.

		**Total body weight (g)**	**Testes weight (g)**	**GSI %**
Initial weight		1,256.7 ± 40.9f	–	–
One month	Control	1,756.7 ± 57.3e	4.93 ± 0.27e	0.280 ± 0.02c
	One dose	1,990.0 ± 44.8d	6.14 ± 0.43d	0.309 ± 0.03c
	Two doses	–	–	–
Two months	Control	2,256.7 ± 63.6c	6.61 ± 0.22c	0.293 ± 0.03c
	One dose	2,696.7 ± 87.4a	9.90 ± 0.95b	0.367 ± 0.03b
	Two doses	2,960.0 ± 99.5b	14.04 ± 1.31a	0.474 ± 0.04a

*Values are mean ± S.E. Means with different letters in each column are significantly different (P < 0.05)*.

**Table 3:** Serum testosterone level (ng/ml) in control and L. tyrosine-treated New Zealand bucks.

	**0 day**	**2 w**	**4 w**	**6 w**	**8 w**	**10 w**
Control	0.250 ± 0.05F	0.470 ± 0.17bE	0.663 ± 0.09bD	0.817 ± 0.09cC	1.143 ± 0.11cB	2.120 ± 0.12cA
One dose	0.266 ± 0.06F	0.833 ± 0.09aE	1.117 ± 0.11aD	1.550 ± 0.17bC	2.190 ± 0.21bB	3.177 ± 0.23bA
Two doses	0.267 ± 0.05F	0.813 ± 0.09aE	1.163 ± 0.11aD	1.970 ± 0.18aC	2.580 ± 0.24aB	3.900 ± 0.36aA

*Values are mean ± S.E. W, weeks after treatment. Means with different small letters in the same column are significantly different (P < 0.05). Letters with different capital letters in the same row are significantly different (P < 0.05)*.

**Table 4:** Serum T_3_ level (ng/dl) in control and L. tyrosine-treated New Zealand bucks.

	**0 day**	**2 w**	**4 w**	**6 w**	**8 w**	**10 w**
Control	107.3 l ± 10.8E	146.3 ± 13.5bD	183.3 ± 17.3bC	215.3 ± 13.5bB	240.3 ± 12.8cA	241.0 ± 22.6cA
One dose	111.0 l ± 9.8D	190.0 ± 13.8aC	289.7 ± 20.6aB	343.0 ± 27.1abA	353.3 ± 20.3bA	303.3 ± 19.5bAB
Two doses	108.7 l ± 11.0D	183.3 ± 15.2aC	285.0 ± 17.5aB	369.7 ± 16.4baA	390.7 ± 27.1aA	354.0 ± 22.3aAB

*Values are mean ± S.E. W, weeks after treatment. Means with different small letters in the same column are significantly different (P < 0.05). Letters with different capital letters in the same row are significantly different (P < 0.05)*.

**Table 5:** Serum T_4_ level (μg/dl) in control and L. tyrosine-treated New Zealand bucks.

	**0 day**	**2 w**	**4 w**	**6 w**	**8 w**	**10 w**
Control	1.22 ± 0.07D	1.52 ± 0.09bC	1.76 ± 0.12cB	2.01 ± 0.16cA	1.98 ± 0.14cA	1.73 ± 0.15cB
One dose	1.22 ± 0.12E	2.46 ± 0.16aC	2.94 ± 0.23bA	2.71 ± 0.15bB	2.52 ± 0.22bC	2.21 ± 0.21bD
Two doses	1.21 ± 0.09E	2.51 ± 0.11aD	3.47 ± 0.19aC	4.27 ± 0.31aA	3.97 ± 0.18aB	3.48 ± 0.24aC

*Values are mean ± S.E. W, weeks after treatment. Means with different small letters in the same column are significantly different (P < 0.05). Letters with different capital letters in the same row are significantly different (P < 0.05)*.

**Figure 1:**
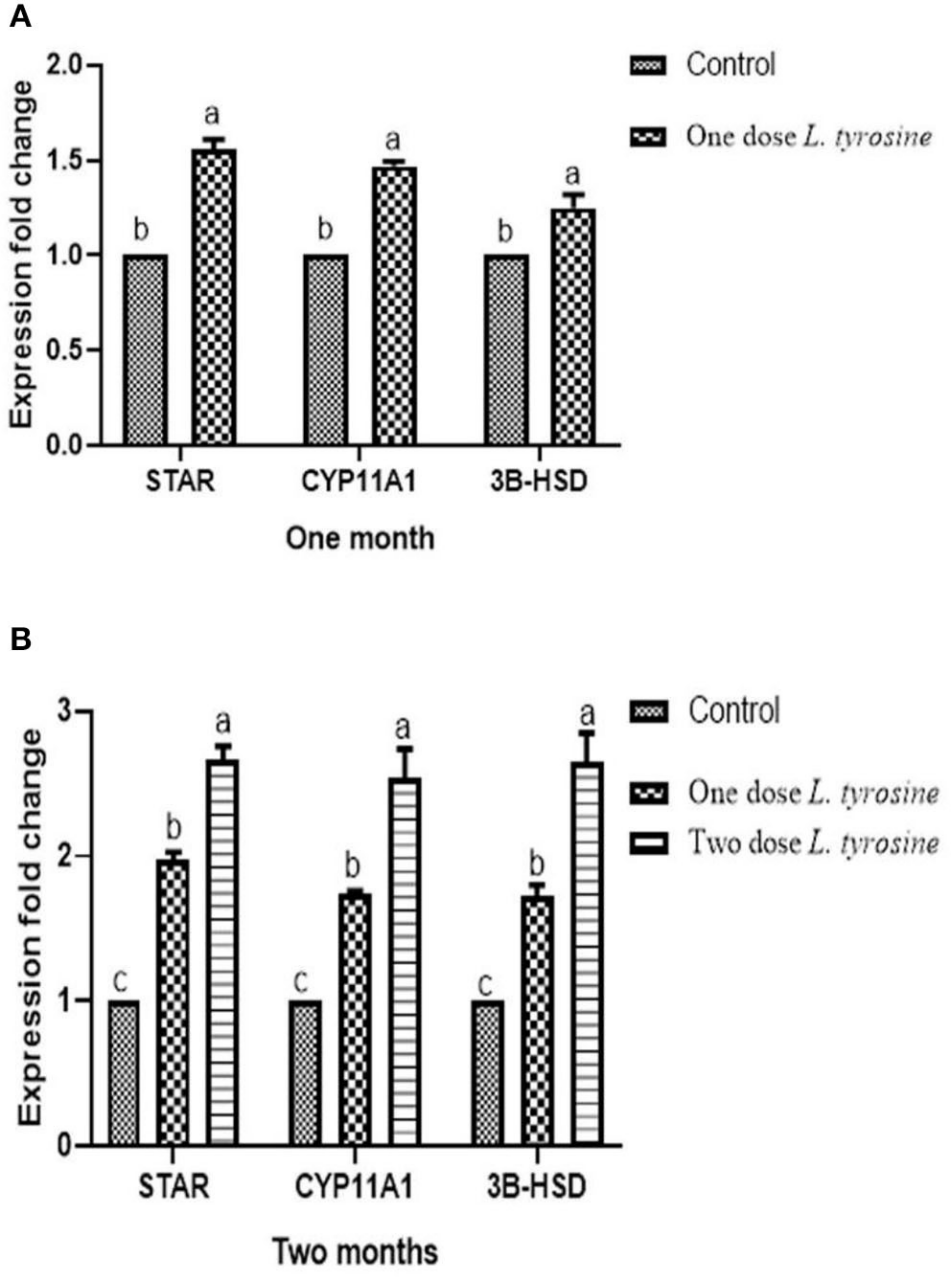
Expression of fold changes of rabbit testicular tissue STAR, CYP11A1, and 3B-HSD mRNA levels after 1 **(A)** and 2 **(B)** months. Data were analyzed with ANOVA followed by Tukey's multiple comparison test. *P* < 0.05, Error bars represent mean ± SD.

**Figure 2:**
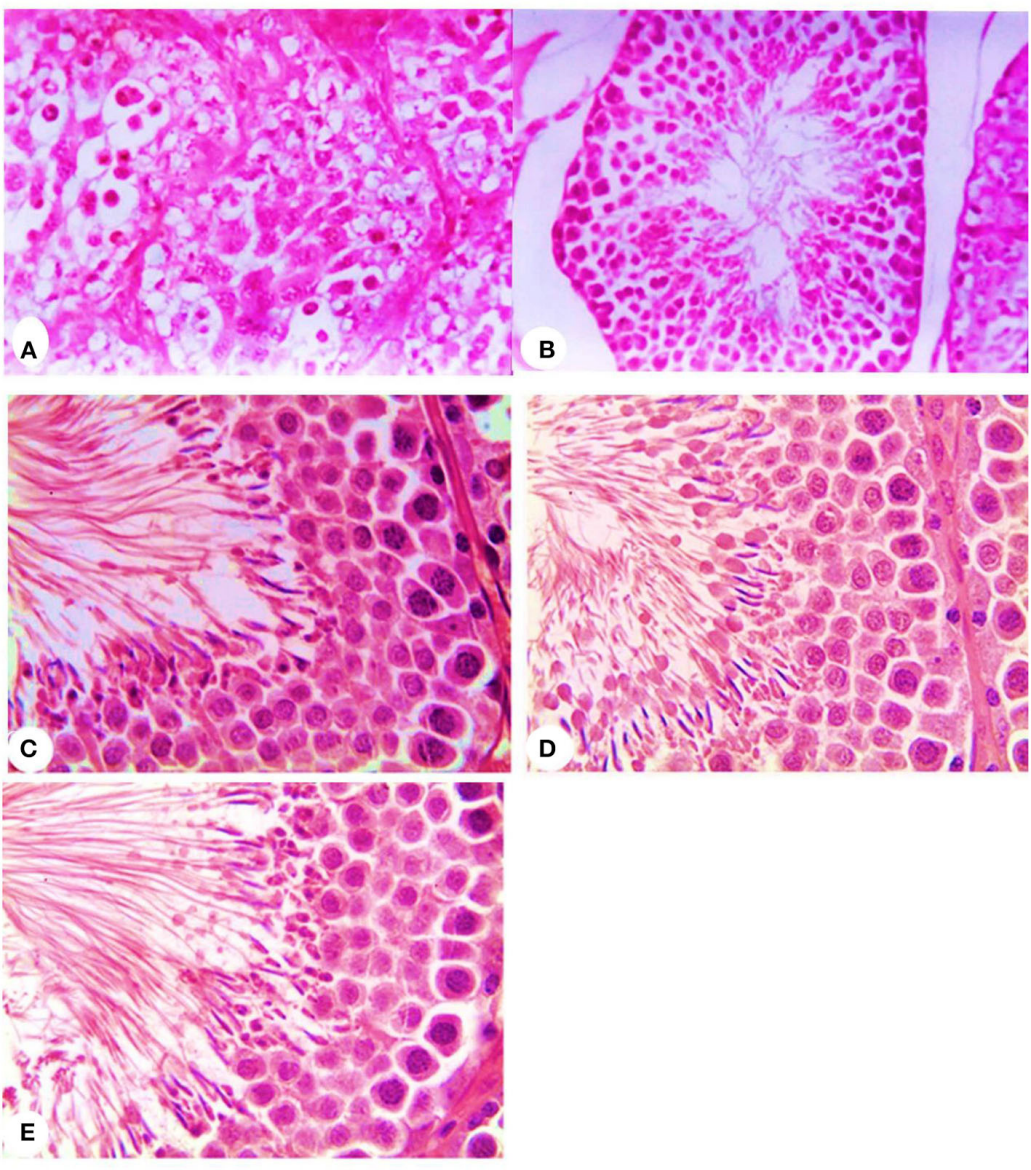
Histopathology of rabbit testicular tissues after 1 month from the beginning of the experiment in control, **(A)** and L-tyrosine treated New Zealand rabbit groups **(B)** and after 2 months from the beginning of the experiment in control, **(C)** and one-dose L-tyrosine **(D)** and two-dose of L-tyrosine treated New Zealand rabbits **(E)** (H & E × 400).

Concerning the reproductive features of New Zealand rabbit bucks, it was observed that testis decline was seen in the control group toward the end of the third month period and 15 days earlier in orally administered L-tyrosine. Semen ejaculate was initially gathered from bucks who received L-tyrosine in the middle of the second month and toward the end of treatment (4 months of age), while, the monitoring control group emerged 1 month apart. The L-tyrosine-dose-dependent rise in semen quantity, cell count, live sperm %, and motility was triggered, with a substantial decrease in reaction point time, and the % of total sperm anomalies were observed in comparison with the control group (Table 6). Steroidogenic enzymes are essential factors in the biosynthesis of various steroid hormones, expression of fold changes of STAR, CYP11A1, and 3B-HSD mRNA in testicular samples of control, and the L-tyrosine treated group is illustrated in Figure 1 in which L-tyrosine substantially (*P* < 0.05) upregulated the expression of STAR, CYP11A1, and 3B-HSD mRNA when compared with the control group especially after 2 months of L-tyrosine treatment. The control rabbits at 3 months of age (1 month from the beginning of the trial) showed seminiferous tubules with developmental stages in which spermatogenesis only reached the spermatocyte stage while the L-tyrosine one dose-treated New Zealand rabbits showed free spermatozoa in seminiferous tubules lumens (Figures 2A,B). After 2 months, each group, control, L-tyrosine one-dose, and L-tyrosine two-dose showed normal spermatogenesis within a normal seminiferous tubule, as shown in Figures 2C–E.

**Table 6:** Seminogram and reaction time in control and L. tyrosine-treated New Zealand bucks.

	**2**^****nd****^ **months post treatment (April)**			**3**^****rd****^ **months post treatment (May)**			**4**^****th****^ **months post treatment (June)**		
	**Control**	**One dose**	**Two doses**	**Control**	**One dose**	**Two doses**	**Control**	**One dose**	**Two doses**
Volume (ml)	No semen	0.9 ± 0.034d	1.1 ± 0.077c	0.7 ± 0.044e	1.2 ± 0.071c	1.6 ± 0.093a	0.7 ± 0.06e	1.1 ± 0.034c	1.4 ± 0.110b
Sperm count(million/ml)	No semen	569.7 ± 11.17c	680.0 ± 23.4a	380.0 ± 19.8e	490.0 ± 24.5d	620.0 ± 24.7b	310.0 ± 11.4f	470.0 ± 17.9d	590.0 ± 20.3bc
Mass motility (0-5)	No semen	3.80 ± 0.07c	4.20 ± 0.09ab	3.20 ± 0.09d	3.80 ± 0.11e	4.33 ± 0.08a	3.40 ± 0.10d	4.00 ± 0.19bc	4.03 ± 0.15bc
Individual motility (%)	No semen	82.0 ± 3.6a	92.7 ± 6.6a	72.0 ± 2.9bc	85.7 ± 5.1a	89.0 ± 4.3a	70.0 ± 3.6c	82.3 ± 5.6ab	88.0 ± 7.2a
Alive sperm (%)	No semen	87.0 ± 3.3ab	90.7 ± 4.3a	80.7 ± 2.8bc	90.0 ± 5.1a	92.7 ± 3.8a	77.0 ± 2.9c	88.0 ± 4.1a	92.0 ± 5.0a
Total abnormality	No semen	5.3 ± 0.27bc	4.7 ± 0.22cd	9.1 ± 0.34a	5.1 ± 0.21c	4.3 ± 0.19b	9.7 ± 0.62a	5.9 ± 0.31b	4.80 ± 0.16cd
Reaction time (sec.)	No semen	55.0 ± 2.2bc	42.0 ± 3.7cd	99.0 ± 4.2a	50.3 ± 1.9cd	40.0 ± 2.1d	95.0 ± 7.2a	65.0 ± 5.8b	49.7 ± 3.2cd

*Values are mean ± S.E. Means with different letters in each raw are significantly different (P < 0.05)*.

## Discussion

The reduction in reproduction and productivity during the summer months is one of the concerns facing rabbit farming. It is important to consider solutions to this problem. To overcome this issue, L-tyrosine is one of the most popular parties for analysis. The blood-borne metabolite amino acid tyrosine may be used to stimulate the release of GnRH since the availability of tyrosine affects norepinephrine synthesis (27, 28).

In this article, L-tyrosine caused a significant dose-related rise in overall body weight (Table 2) and plasma T3 and T4 levels (Tables 4, 5, respectively). Such findings followed Abo Elroos (14) and Omara et al. (15) who verified increases in the T3 and T4 serum levels with age progressing, and L-tyrosine caused an overlaid upsurge in these values. Tyrosine serves as a development-enhancing agent by stimulating hypothalamic catecholamine, dopamine, which stimulates growth hormone release and the thyroid-stimulating hormone (12), as well as the creation of thyroid hormones and protein (29). The activity of the thyroid gland corresponds to body weight since it is accountable for metabolism. Significantly increased body weight demands more mitochondrial energy consumption, with decreased needs for more thyroid hormones (15, 30). Additionally, thyroid secreting hormones promote feed, improve development, and metabolism. Furthermore, thyroid secreting hormones induce other gland secretions and increase the tissue's needs for hormones (31). Increased tyrosine given in this research will be confirmed by intensifying TSH quantity and promoting the discharge of T3 and T4 by the thyroid gland (29). Moreover, Koritschoner et al. (32) reported that thyroid hormones were significant metabolism regulators, and thyroid status faults are often related to altering body weight. Besides, L-tyrosine has useful antioxidant ability-blocking redox oxygen species (33). This can explain the rise in body weight following L-tyrosine supplementation.

Abo Elroos (14) monitored that the pars distalis of the L-tyrosine treated rabbit showed improvement in the quantity of somatotrophs, lactotrophs, and L.H. secreting cells that release GH, prolactin, and gonadotropin, respectively. Consequently, L-tyrosine induces the secretion of GH, TSH, and GnRH, which are also implicated in improving metabolism and body weight and increasing gonad function. Gonadotropin hormones would boost the release of testosterone. The decline in T3 and T4 levels after 4 months of age was attributed to a negative association between temperature and the thyroid gland (May month). Keeping in mind that ensuring the testes descend into the scrotum and the completion of testicular growth, indicating that the testes are beginning to work (9). Clinical findings in this study have shown that tyrosine-treated rabbits have a higher sperm count in their ejaculate compared to the control group. This finding supported tyrosine treatment that may induce early-onset maturity in buck rabbits as a growth-stimulating factor and enhance GnRH that triggers testicular activity (12, 34).

It is important to note that the size of testes during the experiment seemed surprisingly more significant in the L-tyrosine treated rabbits than in the control group. That confirms the correlation among the testicular weight, body weight, and age (5). Nonetheless, because of the influence of L-tyrosine, the level of importance was superimposed because of age. This may be due to L-tyrosine's value as a growth hormone and TSH, T4, and protein synthesis stimulant release factor (29). This was evident in this analysis where L-tyrosine gave rise to GSI and histopathologic findings displaying free spermatozoa in the seminiferous lumen. Also, the spermatocyte point was achieved in the control group. Regarding the seminogram and serum testosterone level, it was apparent that L-tyrosine caused a substantial rise in testosterone and semen ejaculate production, semen quality, and quantity with decreased overall anomalies compared to control. Hall et al. (11) observed a linear association among plasma concentrations and hypothalamic tyrosine levels. L-tyrosine is the catecholamine precursor that triggers the GnRH through the adrenergic receptor pathway (17). GnRH motivates gonadotropin hormones secretion that promotes testosterone secretion (35).

El-Amrawi (18) reported a rise in the libido of bulls after L-tyrosine administration. The stimulating impact of GnRH on the pituitary gonadotropin helped with the creation of the androgen-binding protein transferrin, and markedly improved the libido as a consequence of increasing androgen. Shishkina (36) documented that the troubling association between the noradrenaline of the brain and the hypothalamic-pituitary-testicular axis is likely to be one of the triggers of related reproductive mechanism changes during domestic selection form of conduct. The improvement in semen quality and quantity later reflects the effect of gonadotropin, testosterone, and adrenaline on the seminiferous tubules which reflects the pathway by which L-tyrosine acts (18).

Considering our findings, the reaction time and the amount of the ejaculate increased after testosterone was injected (37), which increased the sexual activity (38). Farhat et al. (39) quoted that the rise in ejaculate quantity following GnRH treatment may be attributed to testosterone's influence on the secretion of accessory glands. Furthermore, Nasr et al. (40) reported that the reaction point-time, semen ejaculate, and libido of the bulls were increased following treatment with GnRH. Concurrently, El-Amrawi et al. (16) showed that the correlation between testosterone and the total sperm count was high, and the time of reaction was negatively correlated.

In this research, L-tyrosine increased sperm motility. This change could be attributed to L-tyrosine's enhancing effect on GnRH (9, 18, 40).

Regarding the % of sperm viability, L-tyrosine improved the live sperm viability. In harmony with our data, live sperm in azoospermia bulls was increased by GnRH (40). Abo Elroos (14) showed that L-tyrosine acts as a stimulating key for the hormone, and connects the hypothalamus and testes.

The findings of this analysis have shown that semen defects in rabbits treated with L-tyrosine declined. That result matches with Idris (37), who reported a decline in primary cell defects after testosterone treatment. Conversely, Nasr et al. (40) stated no sperm abnormalities discovered under a GnRH dose. Experimental outcomes thus suggested that the expression of fold changes of STAR, CYP11A1, and 3B-HSD mRNA in testicular samples of control, and the L-tyrosine treated group in which L-tyrosine substantially upregulated the expression of STAR, CYP11A1, and 3B-HSD mRNA when compared with the control group especially after 2 months of L-tyrosine treatment. The potent L-tyrosine effect appeared after 1 month of treatment concerning the control group, and it was performed after 2 months of L-tyrosine treatment. It was advocated that STAR, CYP11A1, and 3B-HSD were predominantly expressed in gonads (41). Such enzymes are responsible for steroidogenesis, which aims at the formation of endogenous male hormones (testosterone and androstenedione) via different reaction cascades (42). The result obtained from the semen analysis and testosterone and the histological examination of the testes validated our steroidogenic gene research, which endorsed our theory of L-tyrosine significance during the early stages of the sexual activity of the rabbit.

## Conclusion

Oral administration of L-tyrosine could contribute to the increasing weight of the body, improve the sexual quality and quantity of libido and semen in the New Zealand male rabbit bucks, improve their steroidogenesis markers, and enhance early male rabbit sexual growth, which can efficiently be applied during the higher temperature seasons to increase fertility.

## Data Availability Statement

The original contributions presented in the study are included in the article/supplementary material, further inquiries can be directed to the corresponding author/s.

## Ethics Statement

The animal study was reviewed and approved by Faculty of Veterinary Medicine, Kafrelsheikh University.

## Author Contributions

SM, MSa, FF, MA, MD, and MSh: conceptualization, formal analysis, investigation, methodology, project administration, and writing–original draft. All authors: funding acquisition and supervision.

## Conflict of Interest

The authors declare that the research was conducted in the absence of any commercial or financial relationships that could be construed as a potential conflict of interest.
